# Großer Hype um ChatGPT in der Medizin: Ist es etwas für den Rhythmologen? Was muss man bedenken?

**DOI:** 10.1007/s00399-023-00960-5

**Published:** 2023-07-31

**Authors:** W. Haverkamp, N. Strodthoff, J. Tennenbaum, C. Israel

**Affiliations:** 1 Abteilung für Kardiologie und Metabolismus, Medizinische Klinik mit Schwerpunkt Kardiologie, Campus Virchow-Klinikum, Deutsches Herzzentrum der Charité, Charité – Universitätsmedizin Berlin, Augustenburger Platz 1, 13353 Berlin, Deutschland; 2 Berlin Institute of Health Center for Regenerative Therapies (BCRT), Berlin, Deutschland; 3 Department für Versorgungsforschung, Fakultät VI – Medizin und Gesundheitswissenschaften, Abteilung AI4Health, Universität Oldenburg, Oldenburg, Deutschland; 4 Center for the Philosophy of Science, University of Lisbon, Lisbon, Portugal; 5 Klinik für Innere Medizin – Kardiologie, Diabetologie und Nephrologie, Evangelisches Klinikum Bethel, Bielefeld, Deutschland

**Keywords:** Künstliche Intelligenz, ChatGPT, Sprachmodelle, Natural Language Processing, Artificial intelligence, Chat-GPT, Language models, Natural language processing

## Abstract

ChatGPT, ein auf einem großen künstlichen Sprachmodell basierender Chatbot, erregt derzeit viel Aufmerksamkeit. Moderne, auf komplexen neuronalen Netzwerken beruhende Architekturen (sog. Transformer) erlauben ChatGPT, nahezu beliebige Fragen zu beantworten, Zusammenfassungen anzufertigen, zu übersetzen und eigenständig Texte zu generieren. All dies in einem auf Texteingaben basierenden Dialog mit dem Nutzer. Die zugrundeliegenden Technologien mit dem Akronym NLP (Natural Language Processing) gehen in die 1960er-Jahre zurück. In fast allen Bereichen weckt ChatGPT derzeit viele Hoffnungen. Medizinische Examina besteht ChatGPT problemlos; auch über einen Einsatz in Patientenbetreuung, Diagnostik und Therapie und der medizinischen Wissenschaft wird diskutiert. Die fast grenzenlose Begeisterung, die manche Medizinier dieser neuen Technik entgegenbringen, verwundert. Das System weiß zwar enorm viel, aber nicht alles; auch nicht alles, was es ausgibt, stimmt auch. Jede Ausgabe muss auf ihre Richtigkeit hin vom Nutzer sorgfältig kontrolliert werden – oft nicht einfach, da Quellenangaben fehlen. Auch existieren datenschutzrechtliche und ethisch-moralische Probleme. Heutige Sprachmodelle sind nicht frei von Vorurteilen und systematischer Verzerrung. Diese Unzulänglichkeiten haben Stimmen laut werden lassen, die eine stärkere Regulierung des Einsatzes von ChatGPT fordern.

Potenzieller Nutzen und kommerzielle Aspekte dürften dazu führen, dass sich große Sprachmodelle wie ChatGPT zunehmend verbreiten werden. In unserem beruflichen Alltag werden sie immer mehr Platz einnehmen – auch in der Rhythmologie. Zukünftig wird es vor allem darauf ankommen, die Modelle fehlerfreier und transparenter zu gestalten sowie die Regeln für ihre Nutzung klar zu definieren. Eine verantwortungsvolle Nutzung erfordert systematische Schulungen zur Verbesserung der digitalen Kompetenz der Nutzer, einschließlich der Ärzte, die solche Programme einsetzen.

## Abstract (en)

ChatGPT, a chatbot based on a large language model, is currently attracting much attention. Modern machine learning (ML) architectures enable the program to answer almost any question, to summarize, translate, and even generate its own texts, all in a text-based dialogue with the user. Underlying technologies, summarized under the acronym NLP (natural language processing), go back to the 1960s. In almost all areas including medicine, ChatGPT is raising enormous hopes. It can easily pass medical exams and may be useful in patient care, diagnostic and therapeutic assistance, and medical research. The enthusiasm for this new technology shown even by medical professionals is surprising. Although the system knows much, it does not know everything; not everything it outputs is accurate either. Every output has to be carefully checked by the user for correctness, which is often not easily done since references to sources are lacking. Issues regarding data protection and ethics also arise. Today’s language models are not free of bias and systematic distortion. These shortcomings have led to calls for stronger regulation of the use of ChatGPT and an increasing number of similar language models. However, this new technology represents an enormous progress in knowledge processing and dissemination. Numerous scenarios in which ChatGPT can provide assistance are conceivable, including in rhythmology. In the future, it will be crucial to render the models error-free and transparent and to clearly define the rules for their use. Responsible use requires systematic training to improve the digital competence of users, including physicians who use such programs.

Wenn Sie sich darüber informieren möchten, was sich hinter ChatGPT verbirgt, dann fragen Sie das textbasierte Dialogsystem (auch Chatbot genannt) am besten selbst. Dazu gehen Sie ins Internet und geben als URL https://chat.openai.com ein. Hierüber erreichen Sie die englischsprachige Website der ChatGPT betreibenden kalifornischen Firma OpenAI. Nach Anklicken der Schaltfläche „Try ChatGPT“ gelangen Sie auf eine auf den ersten Blick spartanisch gestalte Seite. Unten auf der Seite gibt es ein Feld für die Eingabe von Text. Wenn Sie „Was ist ChatGPT“ eingeben, erhalten nach wenigen Sekunden folgende Antwort: „ChatGPT ist ein großes Sprachmodell, das von OpenAI entwickelt wurde und auf der GPT-Architektur (Generative Pre-trained Transformer) basiert. Es ist darauf trainiert, menschenähnliche Konversationen auf der Grundlage von Texteingaben zu führen. Das Modell wurde mit Millionen von Texten aus dem Internet trainiert und kann in vielen verschiedenen Anwendungen eingesetzt werden, wie zum Beispiel bei der Generierung von Texten, der Beantwortung von Fragen, der Übersetzung von Sprachen und vielem mehr.“ Sie haben auf Deutsch gefragt und bekommen eine deutsche Antwort. Es ist nicht notwendig, Programmeinstellungen zu ändern. Die Sprache wird automatisch erkannt. Wenn Sie ein zweites Mal fragen, ist der Inhalt recht ähnlich, aber die verwendeten Formulierungen unterschieden sich. Unter dem Eingabefeld steht übrigens: „ChatGPT may produce inaccurate information about people, places, or facts.“ Der Satz ist in kleinen Lettern geschrieben; man neigt dazu, ihn zu übersehen.

Die meisten Nutzer sind von dem, was das Programm kann, hellauf begeistert. Es scheint kaum Fragen zu geben, die es nicht in verständlicher Sprache beantwortet. Auch im Bereich der Medizin sieht man zahlreiche Anwendungsmöglichkeiten [2, 6]. Medizinische Examina kann ChatGPT bestehen, und auch über einen Einsatz in der Patientenbetreuung, über Hilfestellung in der Diagnostik und Therapie und über den Einsatz in der medizinischen Wissenschaft wird schon diskutiert [5, 8]. Wenn dem so ist, sollte das Programm nicht auch für Rhythmologen nützlich bzw. hilfreich sein? Dieser Frage und allgemeinen Aspekten der Anwendung von ChatGPT in der Medizin geht die vorliegende Arbeit nach.

## ChatGPT

**Figure 1:**
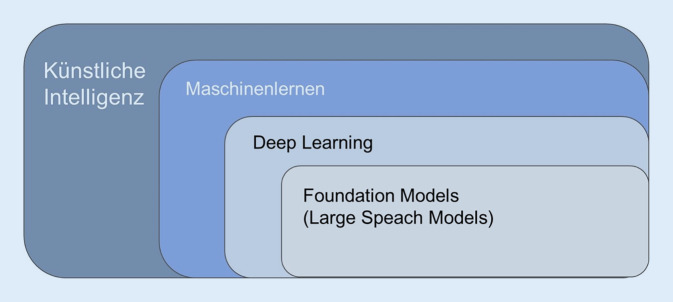

Der Chatbot ChatGPT ist der derzeit bekannteste, seit Ende November 2022 in der Version 3.5 öffentlich frei zugängliche Vertreter einer neuen Generation von auf künstlicher Intelligenz basierenden Computer-Architekturen, die als große Sprachmodelle (Large Language Models, LMMs) bezeichnet werden (Tab. 1; Abb. 1; [9]). Technologische Grundlage ist die Verwendung von Transformern, einer speziellen Architektur von neuronalen Netzen [10]. Das „T“ in ChatGPT nimmt hierauf Bezug. Das, was diese Technologie von rekurrenten neuronalen Netzwerken unterscheidet, die lange den Bereich der computerbasierten Verarbeitung von natürlicher Sprache (Natural Language Processing) bestimmt haben, ist, dass es Daten nicht nur nacheinander (sequenziell), sondern auch parallel verarbeiten kann [10]. Den Kern der Transformer-Architektur bilden diverse, sog. Aufmerksamkeitsmechanismen, die in der Lage sind, Wörter innerhalb eines Textes komplex zu gewichten. Diese Aufmerksamkeitsmechanismen helfen dabei, Zusammenhänge zwischen Wörtern im Text zu analysieren und zu verstehen. Hierdurch gelingt es letztendlich Wort für Wort, basierend auf Wahrscheinlichkeiten, sinnvolle Textausgaben zu generieren. Das „G“ im Namen steht für diese Fähigkeit („generative“). Damit das Ganze effizient geschieht, wird ChatGPT an einer Unmenge von Textdaten *vortrainiert* [11]. Dafür steht das „P“ im Namen („pretrained“). Es ist im Detail nicht klar, welche Daten für das Training on ChatGPT verwendet wurden. Vom Umfang her, dürfte es aber der größte Datensatz sein, der jemals für solche Zwecke eingesetzt wurde. Hierzu gehört z. B. Wikipedia und das Projekt Gutenberg, indem mehr als 60.000 vom Urheberrecht befreite Bücher enthalten sind. Der größte Teil der Trainingsdaten (ca. 80 %) scheint aber aus dem frei zugänglichen Internet zu stammen. Durch dieses erste Training hat sich ChatGPT ein grundlegendes Verständnis von den mathematischen Zusammenhängen zwischen Textobjekten angeeignet, auf das es bei der Beantwortung von Fragen zurückgreifen kann. In einem zweiten Schritt werden Details trainiert (sog- Fine-Tuning; [7]). Inwieweit medizinische Leitlinien berücksichtigt wurden, ist unklar. Auf jeden Fall können die aktuellen Leitlinien aus dem Jahr 2022 nicht berücksichtigt worden sein: Der Stand des Datenkorpus von ChatGPT ist, wie der Chatbot selbst ausführt, September 2021.

**Table 1:** 

*Künstliche Intelligenz*	Künstliche Intelligenz ist die Fähigkeit einer Maschine (eines Computers), basierend auf mathematischen Algorithmen menschliche Fähigkeiten wie logisches Denken, Lernen, Planen und Kreativität zu imitieren
*Maschinelles Lernen*	Überbegriff für Technologien, die in der Lage sind, *künstlich*, d. h. auf mathematischen Prinzipien basierend, Wissen zu generieren
*Deep Learning mit künstlichen neuronalen Netzen*	Teilbereich der künstlichen Intelligenz, der sich methodisch am Aufbau des menschlichen Gehirns orientiert. Mit Hilfe zahlreicher in Schichten angeordneten Knoten (künstlichen Neuronen, die auf mathematischen Funktionen beruhen), sog. künstlichen neuronalen Netzwerken, werden automatisch und ohne manuelle Vorverarbeitung Merkmale und Muster aus Daten extrahiert. Häufig wird überwachtes Lernen eingesetzt, d. h. das neuronale Netz wird anhand von gelabelten Daten (vom Menschen erzeugten Paaren aus Eingangsdaten und zugehöriger Vorhersagegröße) trainiert. Die so entstehenden Algorithmen werden z. B. zur Klassifikation und Datenvorhersage genutzt. Die Leistungsfähigkeit des Netzes hängt von der Anzahl der Schichten, der Anzahl der Neuronen in jeder Schicht, den verwendeten Aktivierungsfunktionen und der Art des Lernalgorithmus ab
*Verarbeitung natürlicher Sprache (NLP, Natural Language Processing)*	Eine Technologie des maschinellen Lernens, die darauf abzielt, Computern die Fähigkeit zu verleihen, menschliche Sprache (in Schrift- oder Sprachform) zu verstehen, zu manipulieren und zu generieren. Hierzu werden Buchstaben, Teile eines Wortes und ganze Wörter in mathematische Repräsentationen umgewandelt (sog. Tokenisierung), um sie KI-Methoden zugänglich zu machen
*Große Sprachmodell (LLMs, Large Language Models)*	Großes neuronales Netzwerk typischerweise mit Milliarden von Parametern, das auf großen Mengen ungelabelter Textdaten vortrainiert wurde. Gelabelte Daten werden zum Fine-Tuning des Modells genutzt

Ein Textverständnis, wie es dem Menschen eigen ist, hat ChatGPT nicht. Der Sachverhalt, dass ChatGPT seine Antworten auf der Basis einer komplex berechneten Wahrscheinlichkeit generiert, dass ein bestimmtes Wort oder eine bestimmte Phrase sinnvoll und passend ist, dürfte auch dafür verantwortlich sein, dass ChatGPT unerwartete, ungewöhnliche oder gar gänzlich falsche Antworten generiert. In diesem Zusammenhang wird auch von *Halluzinationen* gesprochen. Jede Antwort, die ChatGPT gibt, muss vom Nutzer auf ihre Richtigkeit überprüft werden [1].

Die Entwicklung von ChatGPT und sein Betrieb sind übrigens ausgesprochen kostspielig. OpenAI hat in der Vergangenheit eine ganze Reihe von Geldern von diversen Partnern erhalten, darunter auch von Microsoft. Letztere haben kürzlich angekündigt, weitere 10 Mrd. Dollar zu investieren [3]. Die genauen Kosten für den Betrieb von ChatGPT sind nicht bekannt. Es wird jedoch geschätzt, dass die Kosten pro Tag bei etwa 700.000 Dollar liegen [4]. Auf der anderen Seite dürften die Erlöse bzw. der Gewinn für OpenAI erheblich sein. Ende Januar 2023 hatte ChatGPT bereits mehr als 100 Mio. Nutzer weltweit, und es gibt ein Abonnement-Modell, das einen leichteren Zugang und zusätzliche Vergünstigungen bietet und etwa 20 € kostet. Es ist jedoch nicht klar, wie viele Nutzer tatsächlich für ein Abonnement bezahlen und wie viel davon OpenAI als Gewinn verbucht.

## Anwendungsmöglichkeiten

Infobox 1 gibt eine Übersicht über die breit gefächerten Anwendungsmöglichkeiten von ChatGPT in der Medizin [2]. Für alle diese Möglichkeiten lassen sich auch spezielle rhythmologische Anwendungen ableiten. Wenn z. B. ein(e) angehende(r) invasive(r) Elektrophysiologe/in wissen möchte, welche Definition einer speziellen Form von Herzrhythmusstörung zugrunde liegt oder wann und nach welchem Protokoll eine programmierte atriale oder ventrikuläre Stimulation durchgeführt wird, dann lässt sich das einfach erfragen. Die Antwort wird zunächst eher allgemein gehalten sein. Es können jedoch darauf aufbauend weitere Details erfragt werden, z. B. welches die bei der programmierten Stimulation am häufigsten genutzten Stimulationszykluslängen sind. Hierdurch bekommt die Interaktion des Nutzers mit dem Chatbot einen interaktiven Charakter. Es kann auch der Normalwert für die atriale und ventrikuläre Refraktärzeit gefragt werden. Es ist auch möglich, um dazu passende Literaturquellen zu bitten. Man darf sich aber nicht wundern, wenn diese zwar auf den ersten Blick richtig erscheinen, es dann aber doch nicht sind – oder, wenn die DOI, die zum Dokument führen soll, falsch ist und das Anklicken zu einem falschen Dokument führt oder ins Leere geht. Auf die Frage, ob ein Standardwerk der invasiven Elektrophysiologie, verfasst von dem mittlerweile verstorbenen US-Rhythmologen Mark Josephson, Teil des Textkorpus war, der für das Training von ChatGPT verwendet wurde, antwortet das Programm ausweichend mit „it is possible“. Es wird derzeit intensiv diskutiert, wie Urheberrechte bei der Verwendung von Texten für das Training von KI-Modellen wie ChatGPT berücksichtigt werden sollten (bzw. ob diese beim Training von ChatGPT berücksichtigt werden).

Bei Fragen, wie den gerade aufgeführten, funktioniert ChatGPT ähnlich wie eine Internet-Suchmaschine. Nur, dass es nicht eine Liste von Verweisen ist, die sich als Antwort auf die Suche ergibt, sondern ein zusammenhängender, meistens gut verständlicher Text. Microsoft integriert ChatGPT-ähnliche Funktionen gerade in seine Suchmaschine Bing. Auch bei Google ist zu erwarten, dass es in naher Zukunft ChatGPT-ähnliche Funktionen aufweisen wird. In vielerlei Hinsicht gehen die Fähigkeiten von ChatGPT aber über die Möglichkeiten klassischer Suchmaschinen hinaus. Sie können ChatGPT z. B. bitten, einen Untersuchungsbericht abzufassen oder einen Entlassungsbrief zu erstellen, z. B. nach Durchführung einer Katheterablation wegen Vorhofflimmerns. Für Letzteres brauchen Ssie nur Alter und Geschlecht des betroffenen Patienten, die durchgeführte Prozedur eingeben. ChatGPT macht daraufhin einen Vorschlag, der nicht nur allgemeine Aspekte der Katheterablation von Vorhofflimmern, sondern auch Empfehlungen zum Lebensstil und zur Wiedervorstellung beinhaltet. Sie können ChatGPT auch bitten, den Beschwerdebrief eines Patienten zu beantworten. ChatGPT wird Ihnen einen Entwurf liefern. Eine Optimierung eines deutsch- oder englischsprachigen Textes wird ebenfalls innerhalb weniger Sekunden erledigt. Hierzu fügen Sie den Text durch Kopieren und Einfügen in das Eingabefeld ein und bitten um Überprüfung.

## Besonderheiten bei der Anwendung

Die frei zugängliche Version von ChatGPT kann langsam werden oder der Zugang bleibt zunächst blockiert, wenn zu viele Nutzer aktiv sind. Die Abonnement-Version (ChatGPT Plus) ist schneller und erlaubt bereist vorab den Zugang auf neue Versionen. Die praktische Anwendung von ChatGPT muss erlernt werden. Je *besser* die Eingabe ist, desto *besser* wird auch der generierte Text werden. *Besser* bezieht sich hier zum Beispiel auch auf die Eindeutigkeit und die Präzision der Eingabe. Uneindeutige Eingaben erschweren es dem System, klare Antworten bzw. Texte zu generieren. Die Anzahl fehlerhafter Antworten nimmt dann zu. Die Fragen sollten auch nicht zu komplex bzw. kompliziert sein. Hier bietet es sich an, komplizierte Fragen in einfache Fragen aufzuteilen. Ungenaue oder falsche Antworten auf unpräzise Frage können auch dazu führen, dass man gelangweilt die Nutzung aufgibt, ohne das wirkliche Potenzial von ChatGPT erkannt zu haben. Das *intellektuelle* Potenzial von ChatGPT hängt somit von den intellektuellen Fähigkeiten des Nutzers ab.

## Unzulänglichkeiten und Risiken

Infobox 2 führt einige Unzulänglichkeiten und Risiken auf, die bei der Nutzung von ChatGPT berücksichtigt werden müssen [9]. Die Bearbeitung von Eingaben geht bei Sprachmodellen mit einer Vereinfachung der sprachlichen Ausdrucksweise und der Inhalte einher. ChatGPT fällt es nicht nur schwer, sprachlich komplexe Strukturen und/oder Inhalte (längere Sätze, Verwendung von Fremdwörtern, Zahlenmaterial etc.) zu verarbeiten, sondern auch, diese auszugeben. Für die Erstellung wissenschaftlicher Dokumente ist ChatGPT nur sehr eingeschränkt verwendbar. Die allgemeinverständliche Ausdrucksweise des Programms ist somit kein Qualitätskriterium (wie man meinen könnte), sondern eine programmimmanente Notwendigkeit, die mit den Besonderheiten des Modells und dessen Trainings zu tun hat. Details werden oft erst im Zusammenhang mit expliziten Nachfragen ausgegeben. Das kostet Zeit. Dieses Schwafeln des Programms kann auf die Dauer nervig und langweilig werden. ChatGPT stellt vor allem dann, wenn es um Schnelligkeit, Fakten und Zahlen geht, keinen Ersatz für eine übliche Suchmaschine dar.

Nicht oft genug erwähnt werden kann die Notwendigkeit der Überprüfung der Textausgaben auf ihre Sinnhaftigkeit und Richtigkeit durch den Nutzer. Die Ausgaben von ChatGPT dürfen nicht unkritisch übernommen werden [1, 9]. Sehr bedenklich ist auch die Verbreitung von rassistischen Positionen und ethisch-moral fragwürdigen Inhalten, die schon vielfach angemahnt wurde [9]. Verwundern darf dies nicht, da ChatGPT basierend auf mathematischen Zusammenhängen agiert und als Maschine keinerlei eigenes ethisch-moralisches Verständnis hat. OpenAI versucht solche Probleme mit speziellen *Filtern* zu lösen. Ein Filter stellt allerdings aber meistens eine Post-hoc-Lösung dar, die für den Augenblick gemacht ist bzw. zu einer Sache passt. Vordringlich wäre zu verhindern, dass unangemessene und ethisch fragwürdige Inhalte überhaupt kreiert werden. Solche proaktiven Filter dürften allerdings nicht einfach zu etablieren sein, da sie bereits bei der Akquirierung und Auswahl der Trainingsdaten ansetzen müssten. Es stellt sich die Frage, ob und inwieweit die notwendigen, enormen Datenmengen, die zum Training (das übrigens – trotz mehr als 10.000 in einer Cloud zusammengeschalter Computer-Graphikprozessoren – mehrere Monate dauert) überhaupt zu kontrollieren sind.

## Besondere Aspekte der medizinischen Nutzung

Die derzeit auch in medizinischen Zeitschriften berichtete und viel gerühmte Fähigkeit von ChatGPT, ein ärztliches Staatsexamen zu bestehen [4, 8], hält einer kritischen Überprüfung als Kriterium für die Qualität einer künstlichen Intelligenz nicht stand. Auch dem Medizinstudenten im ersten Semester dürfte dies gelingen, sofern man ihn das Internet und eine der üblichen Suchmaschinen nutzen lässt und genügend Zeit gibt. Die Lösung der einzelnen, eher wenig komplexen (meisten auf Multiple-Choice) basierenden Aufgaben ist nicht schwer. Erst die Zusammenstellung zahlreicher Fragen aus unterschiedlichen Disziplinen, unter Ausschluss von Hilfsmitteln und die zeitliche Begrenzung erschweren dem Studenten bzw. der Studentin das Bestehen eines solchen Examens. Letztendlich erstaunt auch die Vielfalt an Informationen, die mit Hilfe von ChatGPT abgefragt werden können. Sie spiegelt den unglaublich großen Umfang an verwendeten Trainingsdaten wider.

Wenn man ChatGPT selbst fragt, ob es sich „im Bereich der Medizin einsetzen lässt“, wird dies wie folgt beantwortet: „[…] ChatGPT kann auch im medizinischen Bereich eingesetzt werden. Da es ein generatives Modell für menschliche Sprache ist, kann es zur Interaktion mit Patienten, zur Beantwortung von medizinischen Fragen, zur Unterstützung bei der Diagnosestellung und zur Entwicklung von Behandlungsplänen eingesetzt werden.“ Vor dem Hintergrund des bereits erwähnten Risikos für Halluzinieren und Konfabulieren, d. h. für die Generierung falscher bzw. fehlerhafter Ausgaben, erscheint diese Aussage sehr bedenklich. Hier überschätzt sich das Programm; potenzieller Schaden ist vorprogrammiert (es haftet übrigens der Nutzer!). Medizinische Entscheidungsfindung, sei es in der Diagnostik oder auch in der Therapie, ist mehr als nur die Erzeugung eines sich natürlich anhörenden Surrogats dessen, was im Internet auffindbar ist. Es gibt keinen Anlass anzunehmen, das ChatGPT unter Berücksichtigung dessen agiert, was in den letzten Jahren eine wichtige Grundlage medizinischer Entscheidungsfindung geworden ist, nämlich einer auf Evidenz basierenden Medizin. Der Prozess der medizinischen Entscheidungsfindung ist weitaus komplexer als die Generierung von auf den ersten Blick sinnvoll erscheinen Texten anhand mathematischer Regeln. Unter anderem fließen wirkliche Einsicht, meist langjährige persönliche Erfahrung und Reflektionen darüber, die Bildung einer eigenen individuellen Herangehensweise und schließlich das Begreifen in größeren Zusammenhängen mit ein. All dies sind bei der medizinischen Entscheidungsfindung oft den Ausschlag gebende Faktoren. ChatGPT besitzt keine dieser intellektuellen Fähigkeiten (hier stimmt ChatGPT übrigens zu). Denkbar ist sogar, dass die häufige und langfristige Nutzung von ChatGPT die gerade aufgeführten ärztlichen Fähigkeiten negativ beeinträchtigen könnte. In der Diagnostik und Therapie zu entscheiden, lernt der Arzt nur, indem er immer wieder, bei jedem Fall erneut, selbst entscheidet.

## Zukünftige Perspektiven

Sprachmodelle wie ChatGPT sind eindeutig ein Fortschritt auf dem Gebiert der künstlichen Intelligenz, der eine große Vielfalt von sinnvollen Nutzungsmöglichkeiten in ganz unterschiedlichen Lebensbereichen und Disziplinen verspricht. Eine Verbesserung der Genauigkeit und Zuverlässigkeit der dargebotenen Informationen dürfte zukünftig erreicht werden [9]. Eine Optimierung der Anpassung an verschiedene Sprachen und Kulturen sollte auch erfolgen. Für die Anwendung solcher Sprachmodelle in der Medizin müssen aber für jede über die Informationsgewinnung hinausgehende Verwendung spezielle Voraussetzungen erfüllt sein, um einen möglichen Schaden von Patienten abzuhalten. Es dürfte z. B. essenziell sein, die Richtigkeit der dargebotenen Information durch einen Quellennachweis zu belegen. Auch die Aktualität der dargebotenen Informationen (z. B. in Form der Berücksichtigung aktueller medizinischer Leitlinien und anderweitiger Empfehlungen) muss gewährleistet sein. Vor dem Hintergrund, dass sich Medizin in einem ständigen Wandel befindet und auch hier die Halbwertszeit des Wissens immer kürzer wird, ist der Stand des Wissens des Chatbots (September 2021) nicht akzeptabel. Für die Zukunft sind große Sprachmodelle, die bezüglich all dieser Aspekte besser sind, gut vorstellbar. Ob diese Modelle tatsächlich irgendwann, in umfassender Form und medizinischem Sinne, zur Verfügung stehen werden, ist unklar. Die Entwicklung und Instandhaltung solcher Systeme, die auch eine fortwährende Aktualisierung beinhalten würde, dürfte enorme Summen verschlingen. Möglicherweise werden viele *kleinere* große Sprachmodelle, die speziellen Zwecken entsprechend trainiert wurden und zertifiziert sind, nebeneinander existieren; eine Entwicklung, die sich jetzt schon abzeichnet.

## Fazit

Chatbots wie ChatGPT, die auf großen Sprachmodellen beruhen, stellen eine spannende Entwicklung dar, die auch im medizinischen Bereich (inklusive der Rhythmologie) in vielerlei Hinsicht hilfreich sein kann. Die Entwicklung ist noch lange nicht abgeschlossen. In weiteren Ausbaustufen werden demnächst auch Bilder (und damit zukünftig vermutlich auch z. B. EKGs und Röntgenbilder) als Eingabe möglich sein. Ein ausschlaggebender Aspekt der Nutzung dürfte aber sein, dass es wohl dabei bleiben wird, dass ChatGPT Ärzten und anderem medizinischem Personal lediglich Hilfestellung bei der Informationsgewinnung und Entscheidungsfindung leisten wird. Ob wir uns als Ärzte auf Anwendungen wie ChatGPT jemals blind verlassen werden können, ist unwahrscheinlich. Die Meinung der Autoren ist nein. Die Frage ähnelt der nach den Möglichkeiten autonomen Autofahrens oder Fliegens. Vermutlich sind es weniger technische Aspekte, sondern ethisch-moralische Aspekte und Fragen, die hier zum Tragen kommen und beantwortet werden müssen.

Vor dem Hintergrund, dass die öffentliche Diskussion über eine mögliche medizinische Anwendung von Sprachmodellen derzeit eher oberflächlich ist, dürfte es für jeden ärztlichen Nutzer sinnvoll sein, sich selbst eine Meinung bezüglich der praktischen Wertigkeit von Sprachmodellen zu bilden. Hierzu ist eine digitale Kompetenz notwendig, die derzeit noch nicht jeder Arzt besitzt. Es gilt, die Nutzung solcher Programme regelrecht zu erlernen. *Blindes Vertrauen* in die menschenähnliche Kommunikation mit Sprachmodellen ist in keiner Weise angebracht. Systematische Schulungen, basierend auf Curricula, dürften weiterhelfen. Eine solche Kompetenz dürfte auch notwendig sein, um Diskussionen mit Patienten, die solche Sprachmodelle zukünftig mit großer Wahrscheinlichkeit vermehrt nutzen werden (möglicherweise auch in Sinne des Erhalts einer *zweiten Meinung* von KI-Seite), standzuhalten.

### Infobox 1 Anwendungsmöglichkeiten von KI-basierten Sprachmodellen wie ChatGPT in der Medizin

- *Textanalyse und -klassifizierung* („text mining and classification“): strukturierte Analyse großer Textmengen (bestehend aus kleinen oder auch großen Dokumenten), wie z. B. elektronische Patientenakten in Krankenhaus- und Praxisinformationssystemen; quantitative Textanalyse für Forschungszwecke
- *Erkennung bestimmter Entitäten* („named-entity recognition“, NER): Erkennung bestimmter Entitäten und deren Kategorisierung (Beispiele für Entitäten: Namen, Diagnosen, ICD-10-Schlüssel, Geburtsdatum)
- *Beantwortung von Fragen* („question answering“): dialogische Informationsgewinnung, basierend auf Sprach- oder Texteingabe; Chatbots mit Spracheingabe dienen z. B. zur automatisierten Anamneseerhebung, Patientenmanagement (z. B. Terminvereinbarung)
- *Automatische Textzusammenfassung* („summarization“): Zusammenfassung von Arztbriefen und anderen Dokumenten
- *Maschinelle Übersetzung* („translation“): Kommunikation in der Praxis bei Sprachbarrieren, Übersetzung von Arztbriefen, Überführung von medizinischer Fachsprache in für Laien verständliche Alltagssprache
- *Textgenerierung*: Befund- und Diagnosekodierung, Befunderstellung, Verfassung von Arztbriefen, Beschwerdebeantwortung

### Infobox 2 Unzulänglichkeiten und Risiken in Zusammenhang mit der Nutzung von Sprachmodellen wie ChatGPT

- Unzulänglichkeiten bei der Richtigkeit, Zuverlässigkeit und Aktualität der dargebotenen Ergebnisse (inkl. des Fehlens eines Nachweises der verwendeten Quellen)
- Unklarer Datenschutz (z. B. fragliche Preisgabe persönlicher oder vertraulicher Daten)
- Verstärkung bzw. Kreierung von Vorurteilen (Verwendung unfairer oder diskriminierender Informationen in den Trainingsdaten)
- Unangemessene und ethisch bedenkliche Inhalte (Verwendung solche Aspekte beinhaltender Trainingsdaten)
- Unklares Potenzial für Manipulation und Missbrauch (bislang fehlen systematische Untersuchungen zu diesen Themen)
